# Ultrasound targeted microbubble destruction-triggered nitric oxide release via nanoscale ultrasound contrast agent for sensitizing chemoimmunotherapy

**DOI:** 10.1186/s12951-023-01776-8

**Published:** 2023-01-30

**Authors:** Yading Zhao, Dandan Shi, Lu Guo, Mengmeng Shang, Xiao Sun, Dong Meng, Shan Xiao, Xiaoxuan Wang, Jie Li

**Affiliations:** Department of Ultrasound, Qilu Hospital of Shandong University, Jinan, 250012 Shandong China

**Keywords:** Ultrasound contrast agent, Nitric oxide, Reactive oxygen species, Hypoxia, Chemoimmunotherapy

## Abstract

Immunotherapy had demonstrated inspiring effects in tumor treatment, but only a minority of people could benefit owing to the hypoxic and immune-suppressed tumor microenvironment (TME). Therefore, there was an urgent need for a strategy that could relieve hypoxia and increase infiltration of tumor lymphocytes simultaneously. In this study, a novel acidity-responsive nanoscale ultrasound contrast agent (L-Arg@PTX nanodroplets) was constructed to co-deliver paclitaxel (PTX) and L-arginine (L-Arg) using the homogenization/emulsification method. The L-Arg@PTX nanodroplets with uniform size of about 300 nm and high drug loading efficiency displayed good ultrasound diagnostic imaging capability, improved tumor aggregation and achieved ultrasound-triggered drug release, which could prevent the premature leakage of drugs and thus improve biosafety. More critically, L-Arg@PTX nanodroplets in combination with ultrasound targeted microbubble destruction (UTMD) could increase cellular reactive oxygen species (ROS), which exerted an oxidizing effect that converted L-Arg into nitric oxide (NO), thus alleviating hypoxia, sensitizing chemotherapy and increasing the CD8 + cytotoxic T lymphocytes (CTLs) infiltration. Combined with the chemotherapeutic drug PTX-induced immunogenic cell death (ICD), this promising strategy could enhance immunotherapy synergistically and realize powerful tumor treatment effect. Taken together, L-Arg@PTX nanodroplets was a very hopeful vehicle that integrated drug delivery, diagnostic imaging, and chemoimmunotherapy.

**Graphical Abstract:**

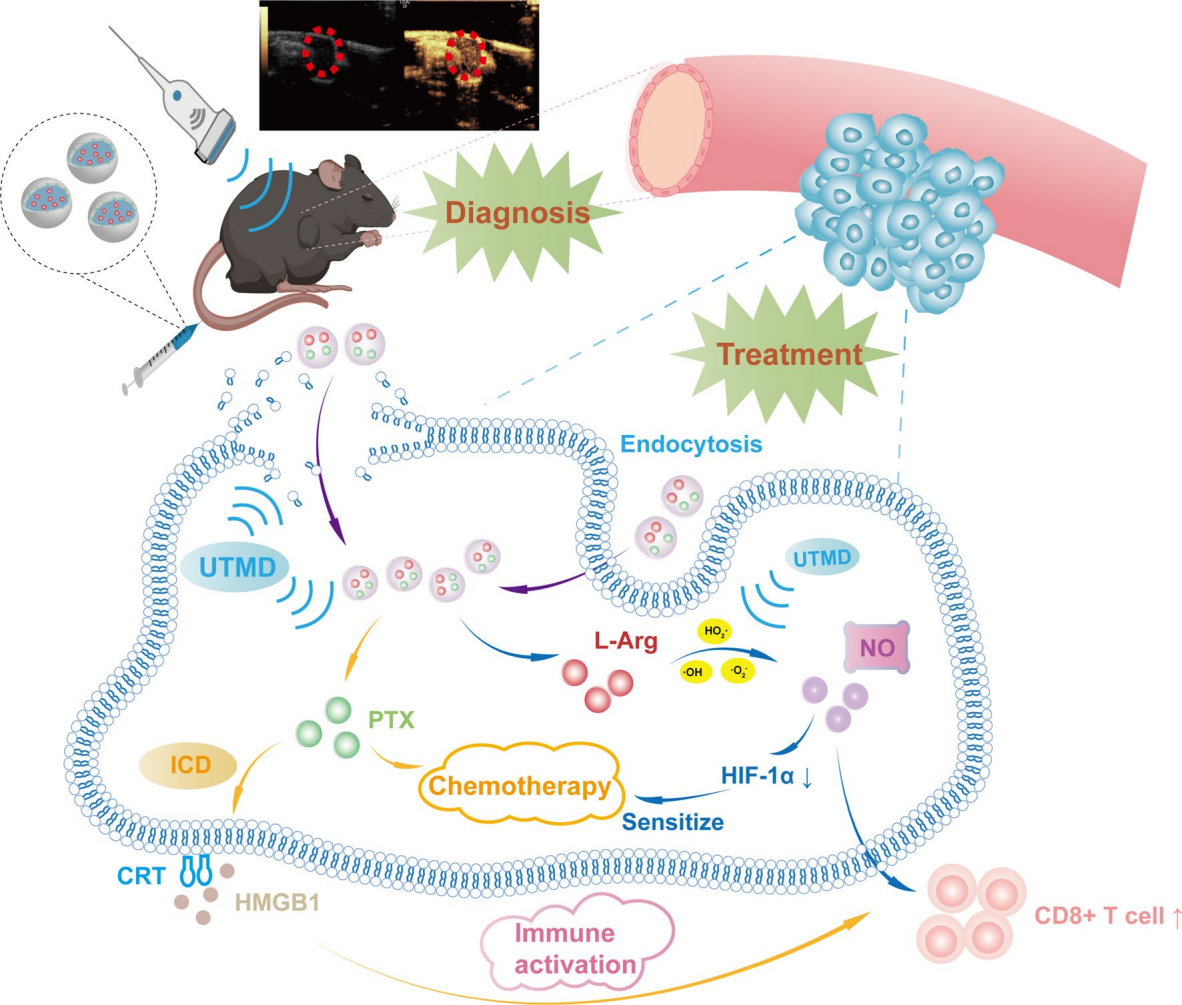

**Supplementary Information:**

The online version contains supplementary material available at 10.1186/s12951-023-01776-8.

## Background

Hepatocellular carcinoma (HCC) is one of the most devastating malignancies in the world [1]. Due to the insidious onset, most patients are usually diagnosed at an advanced stage and are therefore not candidates for surgery and have a very poor prognosis [2]. Recently, immune checkpoint blockade (ICB) has shown great potential to control the progression of advanced HCC [3, 4]; however, the low response rate to ICB remains an important issue [5, 6]. To improve the efficacy, many therapies have been in synergy with immunotherapy. Among them, chemotherapy is considered a suitable candidate for this combination therapy because it can effectively inhibit the growth of tumors [7–9]. More critically, increasing evidences are showing that some chemotherapeutic drugs such as paclitaxel (PTX) could not only cause apoptosis in cancer cells but also induce immunogenic cell death (ICD) to initiate the immune response [10, 11]. Combining chemotherapeutic drug with immunostimulants has gradually shown substantial promise to increase T cell infiltration and antitumor efficacy [12, 13].

Nitric oxide (NO) was an important immunostimulant engaged in vascular regulation and immune response [14, 15]. No induced the expression of endogenous angiogenic factors [16, 17], thereby normalizing tumor vasculature to improve blood perfusion, relieving tumor hypoxia, and regulating the transition from an immunosuppressive to an immune-supportive TME [18]. Nevertheless, its clinical application was hindered by the short half-life and purposeless diffusion behavior [19]. Continuous attempts had been devoted to explore NO donors, such as BNN6 and Nnitrosamines. However, such donors could cause side effects in vivo [20, 21]. Fortunately, L-arginine (L-Arg), with superior biocompatibility, could produce NO catalyzed by inducible NO synthase (iNOS) or reactive oxygen species (ROS) [19, 22, 23]. As for triggers [24–26], light, heat, and X-ray were commonly used, but they were accompanied by some unavoidable drawbacks, such as poor penetration, low controllability and ionizing radiation, seriously compromising their promising applicability for clinical translation. Ultrasound (US) could be a better trigger due to its non-invasive and non-ionizing nature, deeply penetrating ability and highly controllable procedure in increasing intracellular ROS when in conjunction with nanodroplets.

Notably, the immunostimulant NO and chemotherapeutic agent PTX may have separate physicochemical profiles. Therefore, it is a daunting challenge to develop a combined chemoimmunotherapy delivery platform to improve the therapeutic efficacy of HCC. It was commonly believed that nanocarriers could be established for tumor-specific drug delivery, which would enhance the therapeutic effect on tumors and decrease the damage to healthy tissue [27, 28]. Recently, multifunctional nanodroplets, a nanoscale ultrasound contrast agent (UCA), combining diagnostics and visualized treatment had been exploited. The liquid perfluorohexane (PFH)-filled nanodroplets could undergo the liquid–gas phase change to form the large microbubbles. This physical acoustic property made it suitable for contrast-enhanced ultrasound imaging. At the same time, nanodroplets combined with ultrasound offered a prospective platform for delivering drugs in a spatiotemporally controlled way [29]. When nanodroplets were subjected to ultrasound, a sonoporation effect happened, creating pores on the adjacent cell membrane, thus improving cell internalization [30]. Besides, ultrasound-targeted microbubble destruction (UTMD) could lead to accelerated drug release with maximized treatment efficacy. Moreover, given the specific tumor microenvironment (TME) including low extracellular pH, hypoxia, high interstitial fluid pressure [31], nanodroplets should preferably be engineered with stimulus-responsive properties for on-demand targeted drug delivery, which make them a proper candidate to mediate chemoimmunotherapy.

To maximize the antitumor effect, we presented a dual-responsive nanoscale UCA that co-delivered the chemotherapeutic agent PTX and L-Arg for synergistic chemoimmunotherapy of cancer. Here, L-Arg@PTX nanodroplets (NDs) composed of an acidity-responsive O-carboxymethyl chitosan (O-CMC) shell combined with an US-responsive PFH core were successfully constructed and exhibited good biocompatible property. Furthermore, NDs could accumulate specifically in the tumor site and displayed favorable contrast enhanced ultrasound imaging (CEUI) capabilities. Most critically, NDs could release PTX and L-Arg on demand upon UTMD. And, UTMD could produce ROS that catalyzed the release of NO from L-Arg. NO would sensitize PTX-induced chemotherapy through alleviating hypoxia; besides, NO could promote an immune response by activating T cells in synergy with ICD induced by PTX. We are convinced that the L-Arg@PTX nanodroplets could achieve powerful antitumor effect through UTMD-triggered chemoimmunotherapy synergistically.

## Experimental section

### Materials

Tween 20, 0.1% Triton X-100, PTX and L-Arg were obtained from Solarbio (Beijing, China). O-CMC was supplied by Santa Cruz (California, USA). PFH was obtained from Aladdin (Shanghai, China). Lecithin were purchased by Macklin (Shanghai, China). DCFH-DA, DAF-FM DA, DAPI, DiI and DiO were supplied by Beyotime (Shanghai, China). Matrigel was achieved from Corning (New York, USA). Anti-calreticulin (CRT) and anti-high mobility group box 1 (HMGB-1) antibody were supplied by Cell Signaling Technology (Boston, USA). EdU Apollo567 kit was supplied by RiboBio (Guangzhou, China). Anti-CD3-FITC and anti-CD8-PE antibody were supplied by eBioscience (MA, USA). Anti-CD4-APC were supplied by Elabscience (Wuhan, China). ELISA kits were supplied by BOSTER (Wuhan, China).

### Preparation of NDs

The NDs encapsulating L-Arg and PTX were prepared via homogenization/emulsification method [32]. A fixed proportion of PFH (5% v/v), Tween 20 (0.2% v/v) and lecithin (0.13% w/v) were added to deionized water and emulsified by sonicating at 100 W for 5 min (working for 10 s and resting for 10 s) in an ice bath through an ultrasonic cell disrupter (UP-250, Scientz, China). Next, O-CMC solution (2% w/v) with L-Arg and PTX was added dropwise to the mixture for another sonication for 5 min. Then the solution was centrifuged at 300 rpm for 5 min. The middle layer was collected and centrifuged at 13,000 rpm for 15 min. The precipitate was washed with PBS for 3 times to remove free drugs. The purified NDs were resuspended in sterile PBS for further use.

### Characterization of NDs

The morphology of NDs was observed through transmission electron microscopy (TEM). The particle size and zeta potential were examined by Dynamic Light Scattering. High performance liquid chromatography (HPLC) (mobile phase: acetonitrile/water = 55/45 (v/v); flow rate: 1.0 mL/min; wavelength: 227 nm) was applied for determining the amount of PTX encapsulated. Additionally, a microplate reader operating at 570 nm was used for assessing the loading amount of L-Arg that reacted chromogenically with ninhydrin [33–35]. The encapsulation efficiency (EE) and loading efficiency (LE) was set as follows: EE = (Mass of total drug- mass of free drug) / Mass of total drug × 100%; LE = (Mass of total drug- mass of free drug) / Mass of total NDs × 100%.

### Release behaviors of PTX and L-Arg

To investigate drug release behaviors at 37 °C, the NDs performed with or w/o US (1 MHz, 0.5 W/cm^2^, 60 s) were immersed into 1 mL PBS under 100 rpm. At the predefined intervals, the free drug in the supernatant was harvested by centrifugation (13,000 rpm) for measurement.

### Cell culture

The mouse hepatocellular carcinoma Hepa1-6 cells were acquired from Shandong University Cheeloo College of Medicine and cultured in Dulbecco’s modified Eagle’s medium (DMEM) containing 10% fetal bovine serum and 1% penicillin/streptomycin at 37 °C under 5% CO_2_. The cells were treated with various groups: Control, US, PTX + L-Arg + US (Free drug + US), L-Arg@PTX NDs (ND) and L-Arg@PTX NDs + US (ND + US) (10 μg/mL drug, US irradiation: 0.5 W/cm^2^, 60 s, 1.0 MHz). The treated cells were used in the subsequent experiment.

### Cell internalization of NDs

To determine the pH-responsive cell internalization of NDs, DiO-labeled NDs were resuspended in PBS of pH 6.5/7.4. Then, Hepa1-6 cells were cultured in medium containing DiO-labeled NDs for different time. The cells were washed and observed using confocal fluorescence microscopy (Zeiss, Germany). Moreover, the cells were harvested and examined by flow cytometry (FCM, BD, USA).

### In vitro and in vivo CEUI

9L probe of ultrasound diagnostic instrument (GE, USA) was used to implement CEUI. For in vitro CEUI, NDs or PBS was injected into the fingers cut from latex gloves and immersed in degassed water at 37 °C followed by sonication (mechanical index (MI) of 1.0, center frequency of 9.0 MHz, and the dynamic range of 60 dB). For in vivo CEUI, 100 μL of NDs or PBS was administered to the tail vein of mice. Parameters were as follows: MI of 0.8; center frequency of 9.0 MHz; dynamic range of 60 dB. Through ‘Time-Intensity Curve (TIC) analysis’ function of the apparatus, the relative contrast intensity was acquired by subtracting the intensity of the background from that of the region of interest.

### Intracellular ROS and NO release

The Hepa1-6 cells were cultured in 6-well plates overnight. After the treatments, the DCFH-DA ROS kit and the DAF-FM DA NO kit (Beyotime, China) were added, respectively. After 0.5 h, ROS and NO release were detected through fluorescence microscopy and FCM. In addition, nitric oxide concentration was based on measurement of nitrite concentration in culture medium using the Griess reagent by a microplate reader (λ = 540 nm).

### Cell immunofluorescence of CRT and HMGB1

CRT expression on the Hepa1-6 cell membranes was detected by immunofluorescence. In brief, the cells were incubated with various treatments for 24 h, then cells were fixed. When detecting HMGB1, cells were penetrated by 0.1% Triton X-100 after fixation. Cells were incubated with the anti-CRT or anti-HMGB1 at 4 °C, before being subjected to incubation with Alexa Fluor 488-labeled secondary antibody. After dyeing with DAPI, fluorescent images were acquired.

### Western blot of CRT

Membrane protein of the treated cells was obtained through Cell Membrane Protein Extraction Kit following a standard protocol. The proteins were separated by SDS-PAGE and transferred onto 0.22 μm PVDF membranes at 260 mA. Then the PVDF membranes were blocked by QuickBlock^™^ blocking buffer at ambient temperature for 20 min. The membranes were treated with the calreticulin primary antibody at 4 °C overnight. Finally, the membranes were incubated with secondary antibody and visualized using the enhanced chemiluminescence kit.

### ATP release

Cells were subjected to different treatments for 24 h prior to collection of medium for ATP assay. Extracellular ATP was measured with bioluminescence according to a standard protocol and determined using multi-mode board reader (VariosknFlash, Thermo, USA).

### In vitro antitumor effect

The proliferation ability was analyzed through an EdU-567 Cell Proliferation Kit. Cells grown on 6-well plates were subjected to the treatments for 24 h, then they were collected and cultured in 96-well plate. Then, cells were stained according to a standard protocol and were observed by fluorescence microscopy.

The apoptotic ability was detected by Cell Apoptosis Kit. After adherence to 6-well plates, Hepa1-6 cells were subjected to the various stimulations. 24 h later, they were harvested, rinsed, dispersed into binding buffer, as well as stained with FITC and PI. Subsequently, cells were examined by FCM.

The invasion ability was observed by transwell assay. 6 × 10^4^ treated cells dispersed into medium without FBS were grown into an upper chamber containing diluted matrigel, afterwards, medium with 20% FBS was placed in the lower chamber. At the end of 24 h, transwell chambers were dyed with crystal violet. Invasive cells were acquired and reckoned by the microscopy.

### IVIS

Once tumor size reached approximately 300 mm^3^, 100 μL of DiI-NDs was injected intravenously into the tumor-bearing mice. After 5 min, 1 h, 2 h, 4 h, 8 h and 24 h, the mice were anesthetized and observed by in vivo imaging system (IVIS Spectrum, PerkinElmer, USA). Major organs were collected for ex vivo imaging at 24 h. Frozen tumor sections stained by DAPI were imaged using the microscope.

### Animal model

Animal experiments were approved by the Laboratory Animal Ethical and Welfare Committee of Shandong University Cheeloo College of Medicine. Male c57BL/6 J mice (4–6 weeks) with an average body weight of 16–18 g were supplied by Beijing Vital River Laboratory Animal Technology Company and raised in a specific pathogen-free (SPF) environment with a 12 h light–dark cycle. Hepa1-6 tumor-carrying mouse model was developed through injecting 2 × 10^6^ Hepa1-6 cells to the axilla of mouse.

### In vivo antitumor effect

Once the tumor volume reached ~ 150 mm^3^, the mice were randomized to five groups: Control, US, Free drug + US, ND, and ND + US (n = 5). They were treated at day 0, 3, 6, 9, and in the formulations of Free drug + US, ND and ND + US groups, 10 mg PTX and LA/kg mice were supplied. Whereafter, irradiating the tumors with/without ultrasonic probe (1 MHz, 1.5 W/cm^2^) for 60 s after injection of formulations. The tumor volume (length × width^2^ × 0.5) and body weight were recorded every other day. At day 12, the tumors were harvested, weighed and stained with hematoxylin–eosin (HE), TUNEL (an indicator of cell apoptosis) and Ki67 (an indicator of cell proliferation). Moreover, to assess the biosafety, major organs were collected and stained with HE.

### Hypoxia relief and immune response

For immunofluorescence staining, tumor frozen sections were subjected to incubation with blocking buffer for 60 min. Anti-CRT and HIF-1α antibodies were transferred to the slices and incubated at 4 °C. Then, the secondary antibodies were added, followed by nuclear staining. The images were taken by microscope. Meanwhile, after the treatment, the tumors were collected. They were separated to obtain single-cell suspensions, and the suspensions were dyed with anti-CD3-FITC and anti-CD8-PE for cytotoxic T lymphocytes (CD3 + CD8 +), which were then assayed with FCM. Additionally, the cytokines in mice plasma, including IFN-γ and TNF-α, were also examined with ELISA according to a standard protocol.

### Statistical analysis

Experiments were performed at least three time. The data were presented as mean ± SD. Statistical analysis was performed according to the student’s *t*-test or one-way ANOVA using SPSS software (version 23.0, USA). Significance was expressed as *p < 0.05 and **p < 0.01.

## Results and discussion

### Preparation and characterization of NDs

**Fig. 1:**
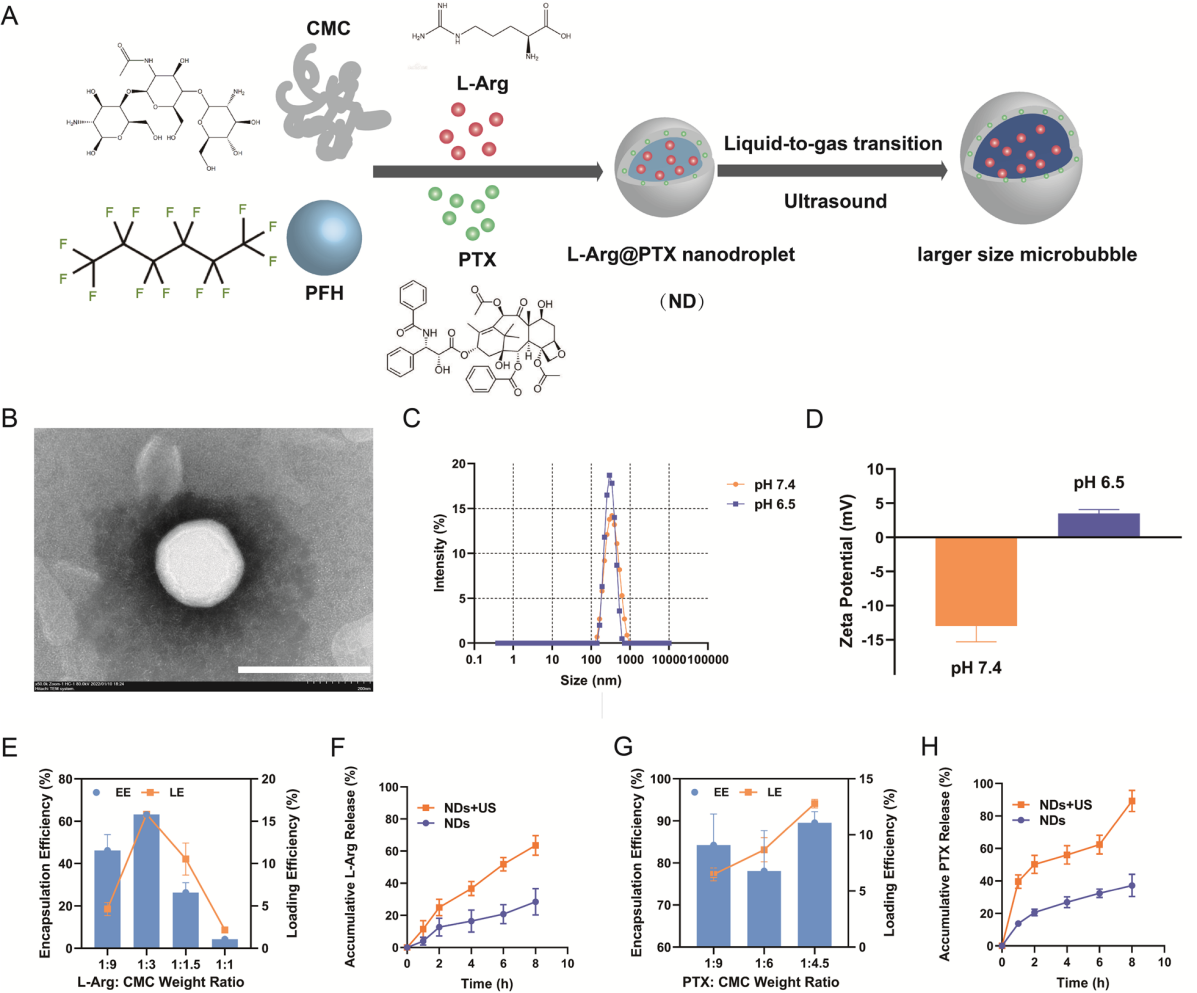
Characterization of NDs. **A** Synthesis diagram of NDs. **B** TEM image of NDs. Scale bar: 400 nm. **C** Size distribution and **D** Zeta potential of NDs under different pH. **E** EE and LE of L-Arg. **F** Drug release behavior of L-Arg under US irradiation. **G** EE and LE of PTX. **H** Drug release behavior of PTX under US irradiation. Data are presented as mean ± SD (n = 3)

To precisely control the release of L-Arg and PTX, we prepared for the first time NDs utilized as a nanocarrier for tumor diagnosis and therapy (Fig. 1A). The morphology of NDs was visualized using TEM. NDs were circular with a clear core–shell structure and a particle size of about 300 nm (Fig. 1B). The pH value of the blood circulation environment was 7.4. However, additional lactate was excreted due to increased glycolytic activity in tumors, leading to the decrease of pH value to 6.5 in TME. The diameter of NDs at different pH values were shown in Fig. 1C. At pH = 7.4, NDs displayed a mean size of 302 ± 11.7 nm. Attributed to their nanoscale size, NDs was able to accumulate locally through enhanced permeability and retention (EPR) effect. Meanwhile, at pH 6.5, the size of NDs was 375 ± 61.8 nm, which was not much different from that at pH 7.4, indicating that NDs were both stable in the physiological environment and TME.

O-CMC, which is composed of both weak base groups (-NH2) and weak acid groups (-COOH) linked by the backbone, regulates the charge conversion of surface in response to environmental pH changes [29]. In the acidic condition, the shell of the nanodroplets (O-CMC) was protonated and the surface charge changed as the pH changed. This characteristic was confirmed by zeta potential conversion of NDs at pH 6.5 and 7.4. The zeta potential was− 13 ± 1.9 mV (pH 7.4) and switched to + 4 ± 0.5 mV at pH 6.5 (Fig. 1D). The negative charge of NDs allowed them to retain in the blood circulation for a long time, while positive charge in TME would increase cellular internalization at tumor sites.

L-Arg and PTX was encapsulated into NDs at an optimal PTX: L-Arg: CMC ratio of 1: 1.5: 4.5 (Fig. 1E, G). EE and LE of L-Arg, PTX at such ratio were 63.1 ± 0.7% and 15.8 ± 0.2%, 89.5 ± 2.2% and 12.8 ± 0.3%, respectively. The drug release profiles of NDs at various conditions were shown in Fig. 1F, H. Small amounts of L-Arg (28.4 ± 6.7%) and PTX (37.2 ± 5.6%) were released from NDs within 8 h, indicating that NDs was stably present in the circulation and not released prematurely. In contrast, the release rate of L-Arg (63.6 ± 5%) and PTX (89.3 ± 5.3%) was significantly quicker under ultrasound irradiation, suggesting the ultrasound-triggered drug release property of NDs. Thus, ultrasound could be employed as a favorable external trigger to improve the efficiency of targeted drug delivery.

### Intracellular NO release triggered by UTMD combined with NDs

**Fig. 2:**
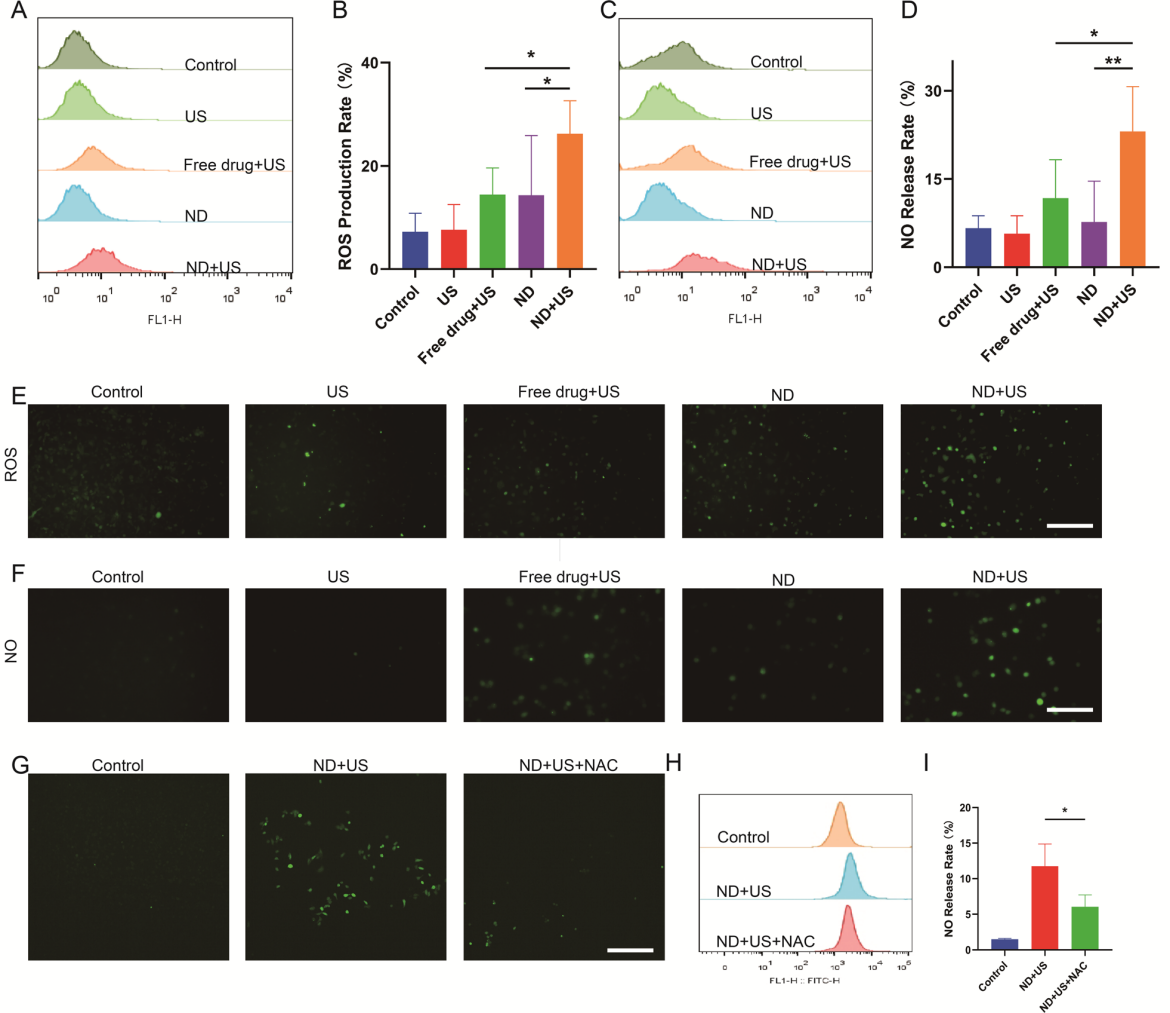
UTMD combined with NDs elicit the release of NO. **A** ROS level after various treatments quantified by FCM. **B** The percentage of ROS production rate obtained from A. **C** NO level after various treatments quantified by FCM. **D** The percentage of NO release rate obtained from C. **E** Fluorescence images of ROS generation (DCFH-DA, green) post different treatments. Scale bar: 200 μm. **F** Fluorescence images of NO release stained with DAF-FM (green) in various treatments. Scale bar: 200 μm. **G** Fluorescence images of NO release (DAF-FM, green) post-treatment (Control, ND + US, ND + US + NAC). Scale bar: 200 μm. **H** NO level post-treatment (Control, ND + US, ND + US + NAC) quantified by FCM. **I** The percentage of NO level obtained from H. Data are presented as mean ± SD (n = 3, *p < 0.05, **p < 0.01)

NO generation was probed through the DAF-FM DA fluorescent probe, which reacted with NO to generate benzotriazole, showing strong green fluorescence. As observed in Fig. 2F, the green fluorescence associated with NO was difficult to be observed in the Control and US groups, and similarly, only a small amount of NO was produced in the ND group without ultrasound; however, when irradiated with ultrasound, the cells showed a more intense green fluorescence, revealing that ultrasound irradiation could effectively induce NO production. Additionally, the stronger fluorescence was detected in the ND + US group compared to the Free drug + US group, indicating that NDs generated more NO compared with free drug under ultrasound stimulation. FCM also showed the same results (Fig. 2C, D).

To verify the mechanism of NO generation, the cellular ROS production was further assessed by the DCFH-DA probe, which reacted with ROS, converting it to green fluorescent 2′,7′,-dichlorofluorescein (DCF) to display green fluorescence. In Fig. 2E, green fluorescence was observed in all groups, and, the strongest fluorescence was displayed in ND + US group, suggesting that UTMD could produce more ROS in the cancer cells, which was in line with previous researches [36]. The FCM data also confirmed the results (Fig. 2A, B). We found that the generation of NO was accompanied by the production of ROS. Therefore, we further explored the relationship between ROS and NO in Fig. 2G, H. We found that the release level of NO was decreased in the ND + US group when pre-treated with NAC (ROS inhibitor) (11.8 ± 2.5% vs. 6.1 ± 1.3%, *p < 0.05) (Fig. 2I). These results confirmed that NO release contained two steps: (1) L-Arg and ROS were released from NDs triggered by UTMD; (2) ROS accelerated the oxidation of L-Arg to NO.

The quantification of NO was performed by a Griess test (Additional file 1: Fig. S2). The NO concentration in the ND + US group was greater than 1 μM at different ultrasound powers and irradiation times. A large number of researchers had reported that NO at high concentrations (> 1 μM) could suppress tumors. Interestingly, the amount of NO in 1.0 W/cm^2^−60 s group was instead reduced compared to the 0.5 W/cm^2^−60 s group, which may be due to the fact that excessive intensity may induce hyperthermia and thus lead to poor cellular status. Therefore, the intensity of 0.5 W/cm^2^−60 s is a relatively mild condition under which NO concentration can be significantly increased, which further indicates that 0.5 W/cm^2^−60 s is a suitable choice in in vitro experiments.

### ICD induced by UTMD combined with NDs

It had been reported that intracellular ROS could remarkably trigger immunotherapy-associated ICD to initiate antitumor immune responses [37, 38]. Simultaneously, studies had demonstrated that PTX could induce ICD. Therefore, the synergistic effect of NDs and UTMD for promoting ICD was furter evaluated. Typical molecules used for validating ICD were CRT exposure on cell surface, HMGB-1 and ATP release, which facilitated anti-tumor immunity [39, 40].

**Fig. 3:**
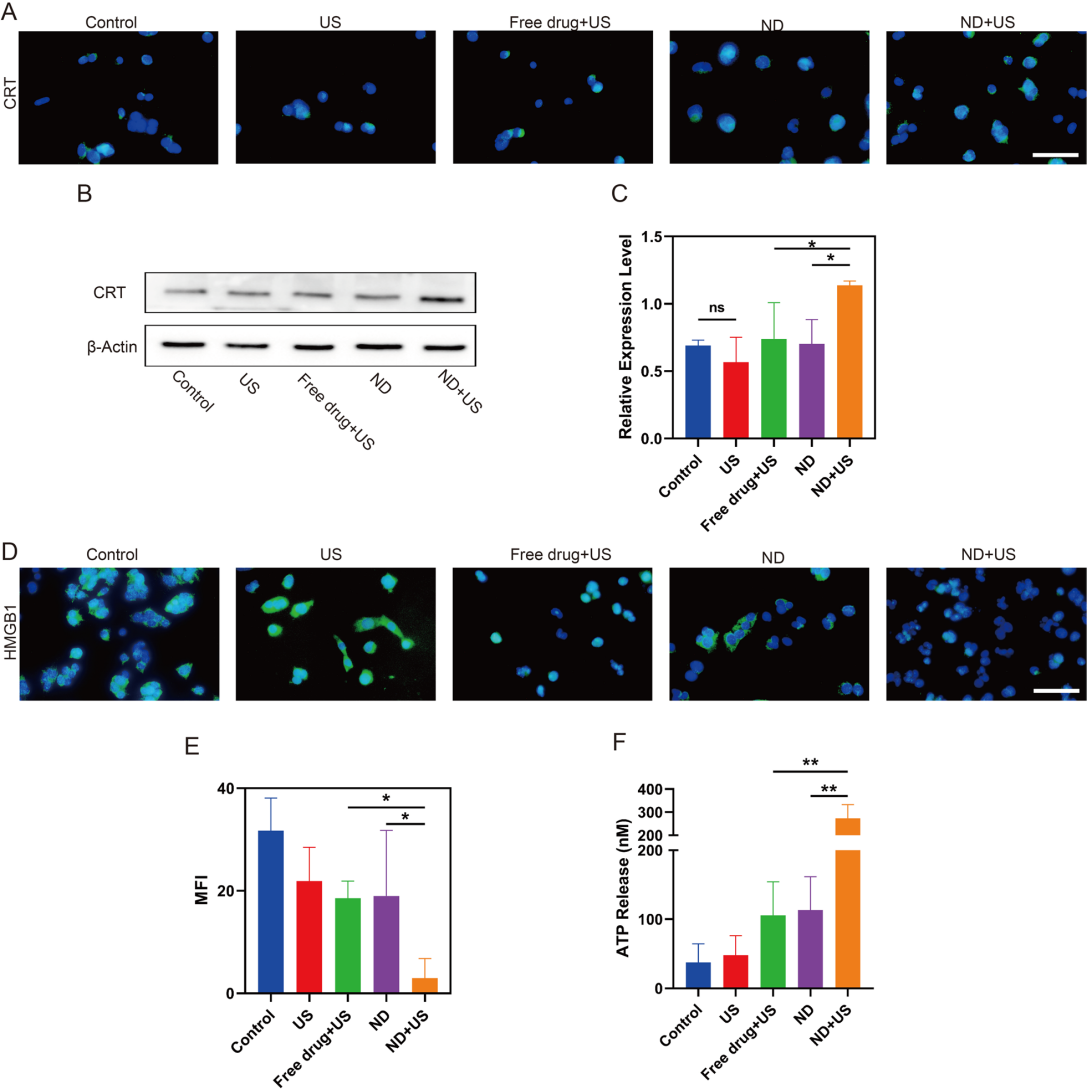
UTMD combined with NDs initiate the ICD.** A** CRT expression of Hepa1-6 cell surfaces post-treatments. The green and blue color represented CRT and cell nuclei, respectively. Scale bar: 50 μm. **B** CRT expression level on the Hepa1-6 cell membranes after various treatments. **C** Quantitative analysis of CRT expression level from B using Image J. **D** HMGB1 expression of Hepa1-6 cells after different treatments. HMGB1 appeared green fluorescence, and cell nuclei stained by DAPI was blue. Scale bar: 50 μm. **E** Semiquantitative analysis of HMGB1 from D using Image J. **F** Extracellular ATP release from Hepa1-6 cells after various treatments. Data are presented as mean ± SD (n = 3, *p < 0.05, **p < 0.01)

Cells stained with CRT antibody after different treatments were observed by fluorescence microscopy. As shown in Fig. 3A, obvious green fluorescence was noted in the ND + US group, with the exception of this group, all other groups showed little CRT expression in the cell surface. The WB results of CRT further showed that the cell surface expression induced by NDs was higher than that of the free drug under ultrasound irradiation (1.1 ± 0.0 vs. 0.7 ± 0.2, *p < 0.05, Fig. 3B, C). In addition, ND + US group-treated cells showed higher expression of CRT than the ND group (1.1 ± 0.0 vs. 0.7 ± 0.1, *p < 0.05). In Fig. 3F, ATP release exhibited similar trends to that of CRT. The translocation of HMGB1 from intracellular to extracellular matrix was observed by immunofluorescence. In Fig. 3D, HMGB1 was mainly located in tumor cells in Control and US groups. Both Free drug + US and ND groups caused moderate release of HMGB1. In contrary, HMGB1 was almost fully released from the cells in the ND + US group. The semi-quantitative results of fluorescence further confirmed this effect (Fig. 3E). Therefore, the results confirmed that ultrasound-mediated L-Arg@PTX-NDs therapy could potently initiate Hepa1-6 cells immunogenic death.

### Accumulation and biodistribution of NDs

**Fig. 4:**
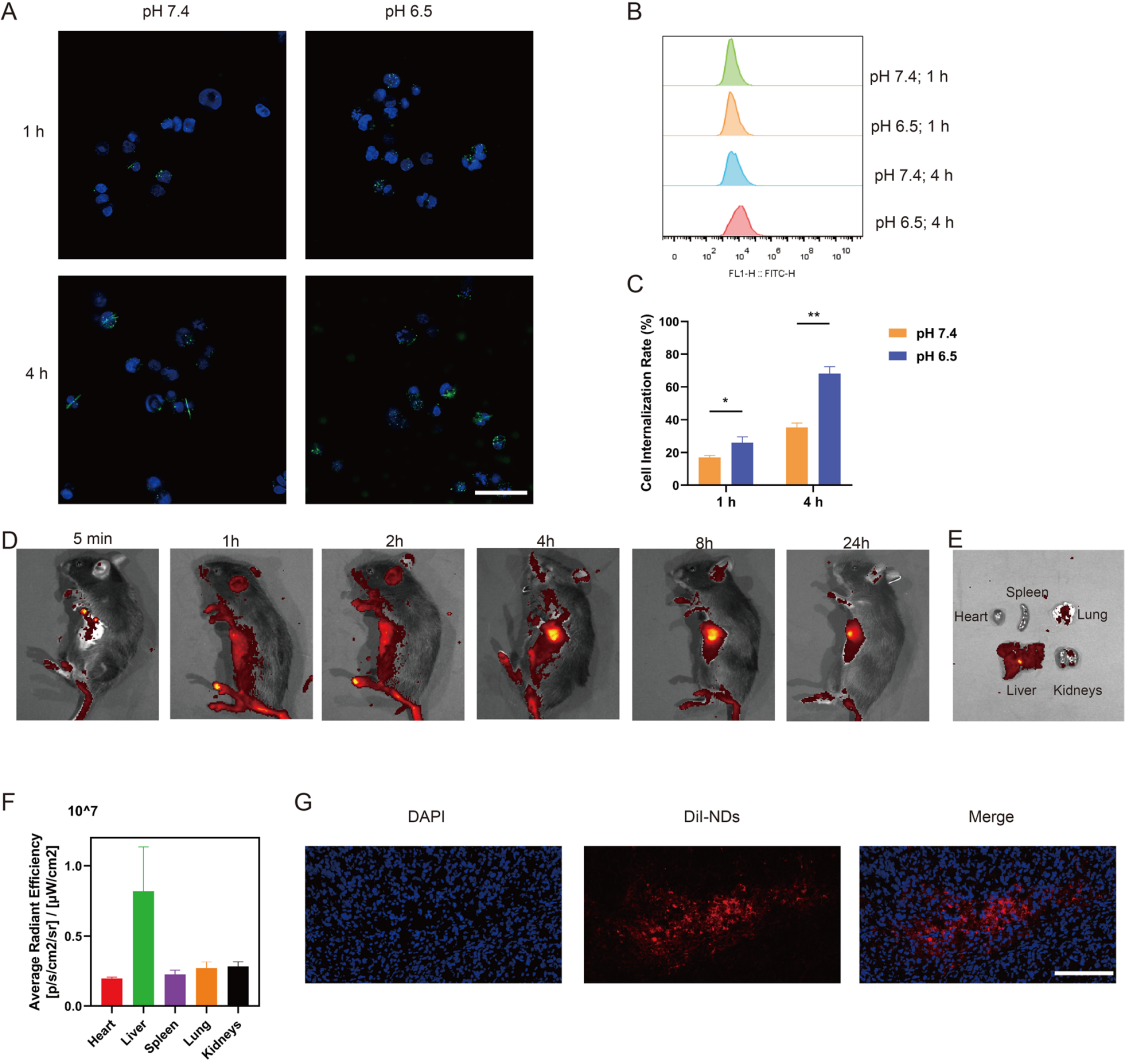
The accumulation and biodistribution of NDs. **A** Cellular uptake of DiO-labeled NDs under different pH values at 1, 4 h. NDs labeled with DiO appeared green dots, and cell nuclei counterstained by Hoechst were blue. Scale bar: 50 μm. **B** FCM of Hepa1-6 cells treated with DiO-labeled NDs under different pH values at 1, 4 h. **C** Cell internalization rate of NDs obtained from B. **D** Representative in vivo images of tumor-bearing mice after intravenous administration of NDs. **E** Representative ex vivo image of main organs at 24 h. **F** Semiquantitative data of fluorescence in major organs from E. **G** The accumulation of NDs at tumors at 24 h. NDs labeled with DiI showed red dots, and cell nuclei stained by DAPI were blue. Scale bar: 100 μm. Data are presented as mean ± SD (n = 3, *p < 0.05, **p < 0.01)

The cell internalization of NDs (labeled with DiO) was assessed via confocal microscope. In Fig. 4A, the green fluorescence around cells increased with time. Remarkably, the green fluorescence at pH 6.5 (tumor microenvironment) was higher than that at pH 7.4 (blood circulation environment). FCM result also revealed that the NDs incubated at pH 6.5 penetrated the cells more quickly and increased over time (Fig. 4B, C). A possible explanation was that NDs achieved charge reversal of O-CMC surface at pH 6.5, facilitating its interaction with negatively charged tumor cell membrane. Even at 12 h and 24 h (Additional file 1: Fig. S3A), numerous NDs aggregated on cells in the pH 6.5 group more than in the pH 7.4 group. The result of fluorescence imaging were corroborated by data from FCM (Additional file 1: Fig. S3B), which showed that NDs incubated at pH 6.5 could also penetrate more cells than NDs incubated at pH 7.4 after 12 h (90.8 ± 0.7% vs. 83.4 ± 0.8%, **p < 0.01) and 24 h (94.1 ± 0.4% vs. 88.7 ± 0.6%, **p < 0.01).

NO exerted a crucial effect on vascular smooth muscle, which enhanced vascular permeability to improve permeability and retention effects and improve accumulation of NDs at tumor site [41]. To ascertain the distribution of NDs in Hepa1-6 tumor-bearing mice, we injected NDs (labeled with DiI) intravenously into the mice and photographed the tumors and main organs at predefined time points. As seen in Fig. 4D, the fluorescence signal of DiI increased over time and reached a peak at 8 h and remained undiminished significantly until 24 h, which suggested that the NO induced by ROS and charge conversion caused by CMC in TME could enhance the accumulation of NDs and realize long-term stable accumulation in the tumor. After 24 h, major organs were harvested and the average radiant efficiency of DiI-labled NDs was obtained, showing a higher intensity in the liver compared to the other organs, due to the metabolism of NDs by the liver (Fig. 4E, F). Moreover, as shown in Fig. 4G, frozen sections of tumor displayed that DiI-labled NDs could be efficiently transferred into the tumor, which may be attributed to the EPR effect and pH responsiveness of NDs.

### CEUI of NDs

**Fig. 5:**
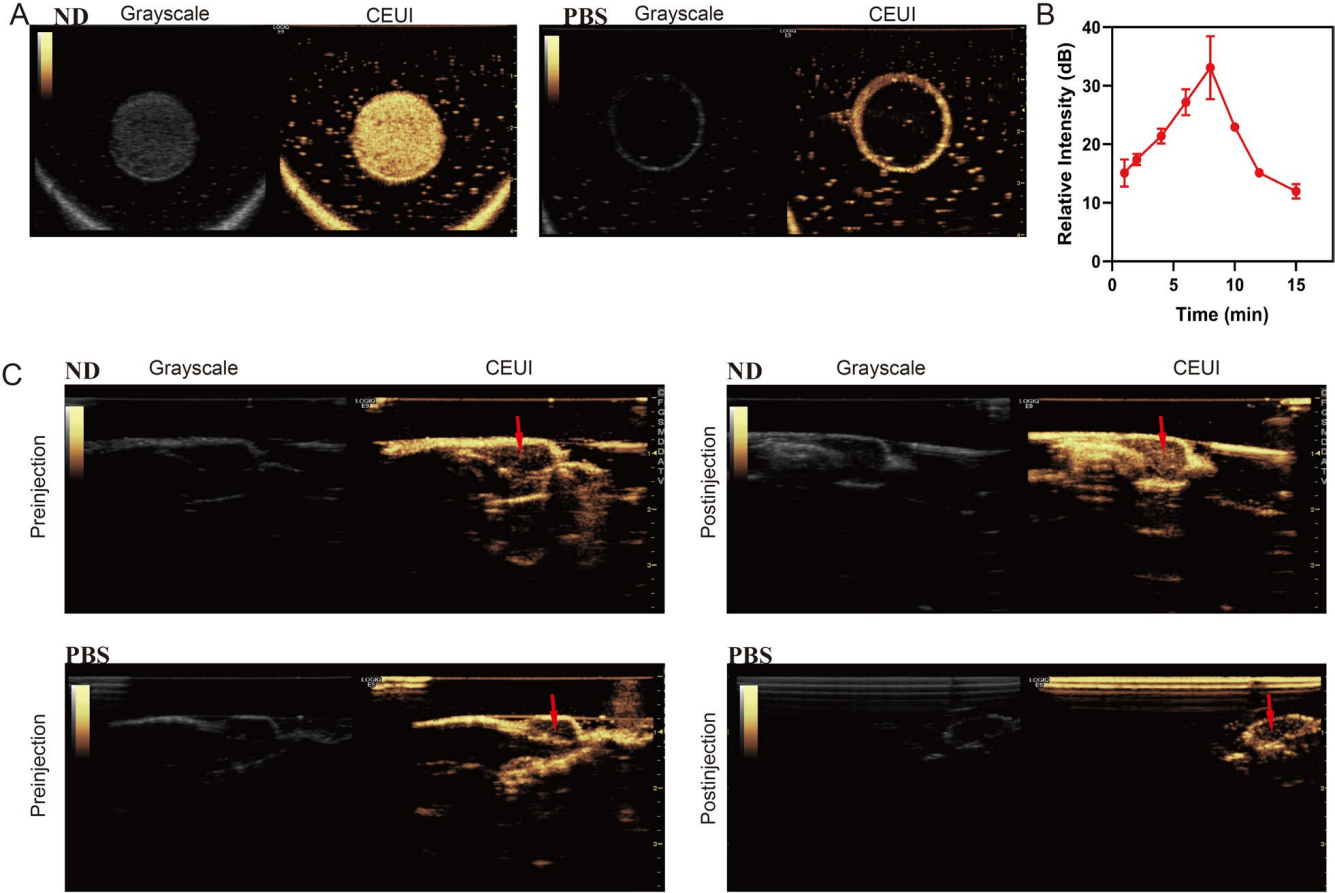
CEUI of NDs. **A** Ultrasound imaging in vitro. **B** TIC in tumors after administration of NDs in vivo. **C** Ultrasound imaging in vivo. The red arrows pointed to the tumor sites

NDs were preferable to common nanosystems due to their ability to be utilized for diagnostic imaging and real-time monitoring in addition to drug delivery therapy. To assess the ultrasound imaging potential of NDs, the in vitro CEUI was conducted. As illustrated in Fig. 5A, no evident echogenic signal was observed in the PBS group. Just the opposite, the echogenic signal of NDs was obviously much stronger with ultrasound exposure.

Figure 5C presented the image of in vivo CEUI, where the tumor showed almost no echogenic signal after PBS administration. In contrast, the NDs group exhibited a marked increase in echogenic signal after administration, and the signal filled the entire tumor, which may be due to the accumulation of NDs at the tumor site. The TIC was acquired by the relative intensity at the tumor at different time after intravenous administration of NDs (Fig. 5B). Of interest, it was found that the echogenic signal of the tumor after NDs administration initially increased over time, greatly enhanced at 8 min, and then gradually decreased, making it beneficial as an unique nanocarrier for diagnostic imaging and therapy.

The liquid (PFH)-core NDs in this study overcame the disadvantages of conventional gas-core nanobubbles with relative large size and suboptimal structural stability. Under ultrasound exposure, the NDs underwent the instantaneous phase change, leading to the formation of large bubbles, which could produce strong contrast with higher acoustic impedance compared to the former NDs [42]. NDs exhibited good imaging capability at 37 °C, a temperature below the boiling point of PFH (58 °C), further proving the dominant role of ultrasound in NDs vaporization. In addition, L-Arg could react with ROS induced by ultrasound to release NO, which may be a cavitation nucleus to enhance CEUI [43].

### In vitro antitumor effect

**Fig. 6:**
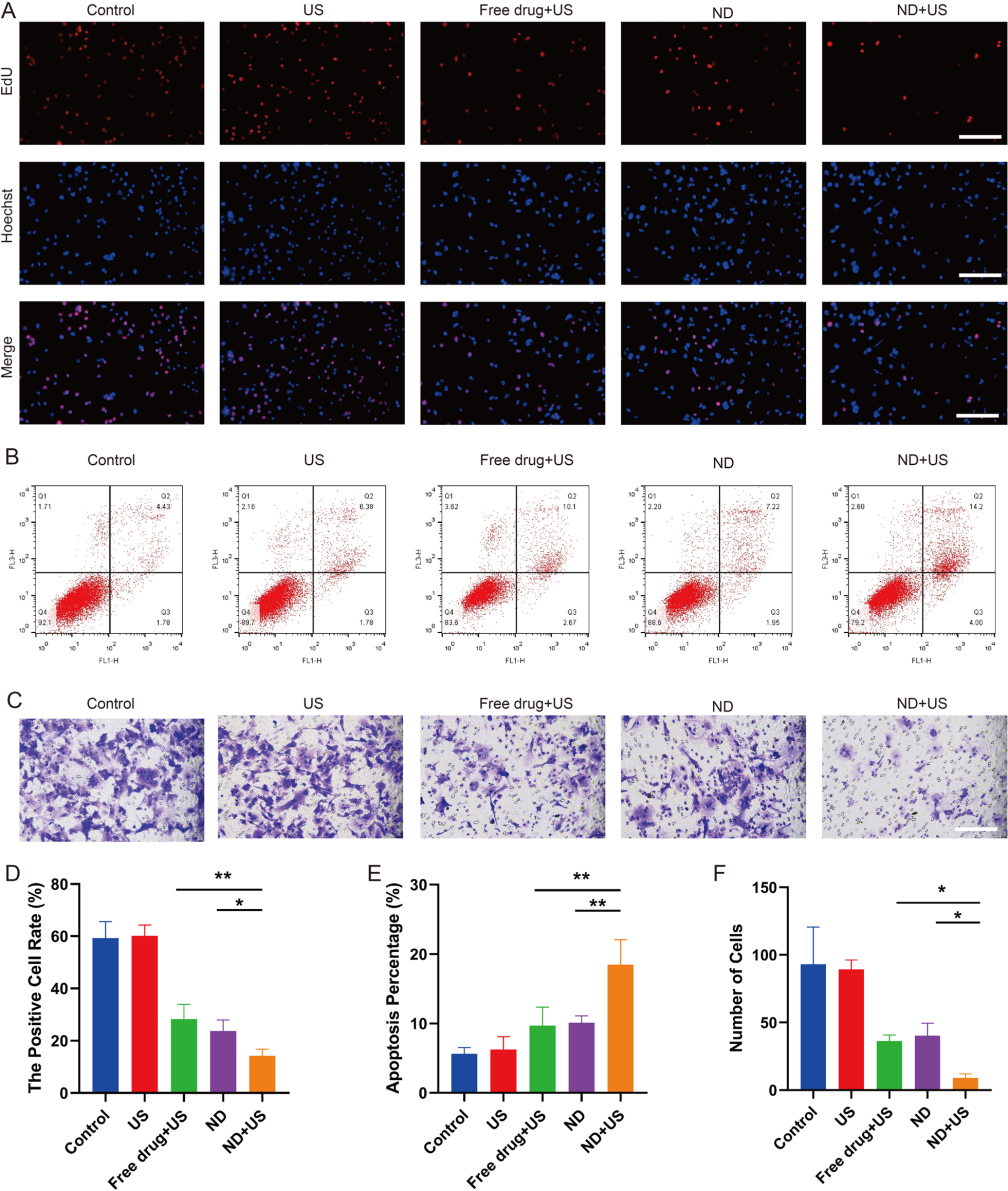
In vitro antitumor effect.** A** Fluorescence images of cell proliferation after various treatments evaluated by EdU assay. Red fluorescence represented proliferative cell, and blue fluorescence represented cell nuclei counterstained by Hoechst. Scale bar: 100 μm. **B** Cell apoptosis by FCM after different treatments. **C** Invasion cells assessed by Transwell assay. Scale bar: 100 μm. **D** The percentage of positive cell rate examined from A by Image J. **E** Quantification of cell apoptosis from **B**. **F** Quantification of the invading cells from **C**. Data are presented as mean ± SD (n = 3, *p < 0.05, **p < 0.01)

The proliferative capacity of the treated cells was shown in Fig. 6A, D, where cells in proliferative phase were dyed red (EdU-positive) and nuclei were dyed blue. A large number of proliferating cells were observed in the US group, demonstrating that ultrasound alone was ineffective in suppressing cell proliferation. Inhibitory effect in ND + US group on cell proliferation was more obvious than that of ND alone (14.2 ± 2.1% vs. 23.8 ± 3.4%, *p < 0.05), indicating that ultrasound irradiation was critical for NDs-mediated therapy. Besides, cell proliferation of ND + US group was lower than that of Free drug + US group (14.2 ± 2.1% vs. 28.3 ± 4.6%, *p < 0.01), indicating that UTMD enhanced the permeability of cell membrane after the NDs were sonicated, which promoted the cellular uptake of drug and thus obviously improved the inhibitory effect [44].

To directly assess apoptosis, Hepa1-6 cells were stained with AnnexinV-FITC/PI (Fig. 6B, E). The total percentage of apoptosis in the Control and US groups was 5.6 ± 0.7% and 6.2 ± 1.5%. In opposite, cell apoptosis was increased in the Free drug + US, ND and ND + US groups, while the number of apoptotic cells was highest in the ND + US group (18.5 ± 2.9%, **p < 0.01), indicating a stronger apoptotic effect than the other treatments. The result revealed that UTMD combined with drug-loaded NDs could significantly promote cell apoptosis.

The invasive ability of Hepa1-6 cells after different treatments was also assessed using a transwell assay. As shown in Fig. 6C, F, the invading cells in Control and US group were much more than any other group. In contrast, the number of invasive cells in the ND + US, Free drug + US and ND groups were 9 ± 2, 36 ± 4 and 40 ± 8, respectively, with the least in the ND + US group (*p < 0.05). The result revealed the effectiveness of NDs under ultrasound irradiation in suppressing cell invasion.

In summary, the described results collectively proved the synergistic effects of the combined strategy of NDs and ultrasound irradiation in inhibiting proliferation, promoting apoptosis and suppressing invasion.

### In vivo antitumor effect and biosafety

**Fig. 7:**
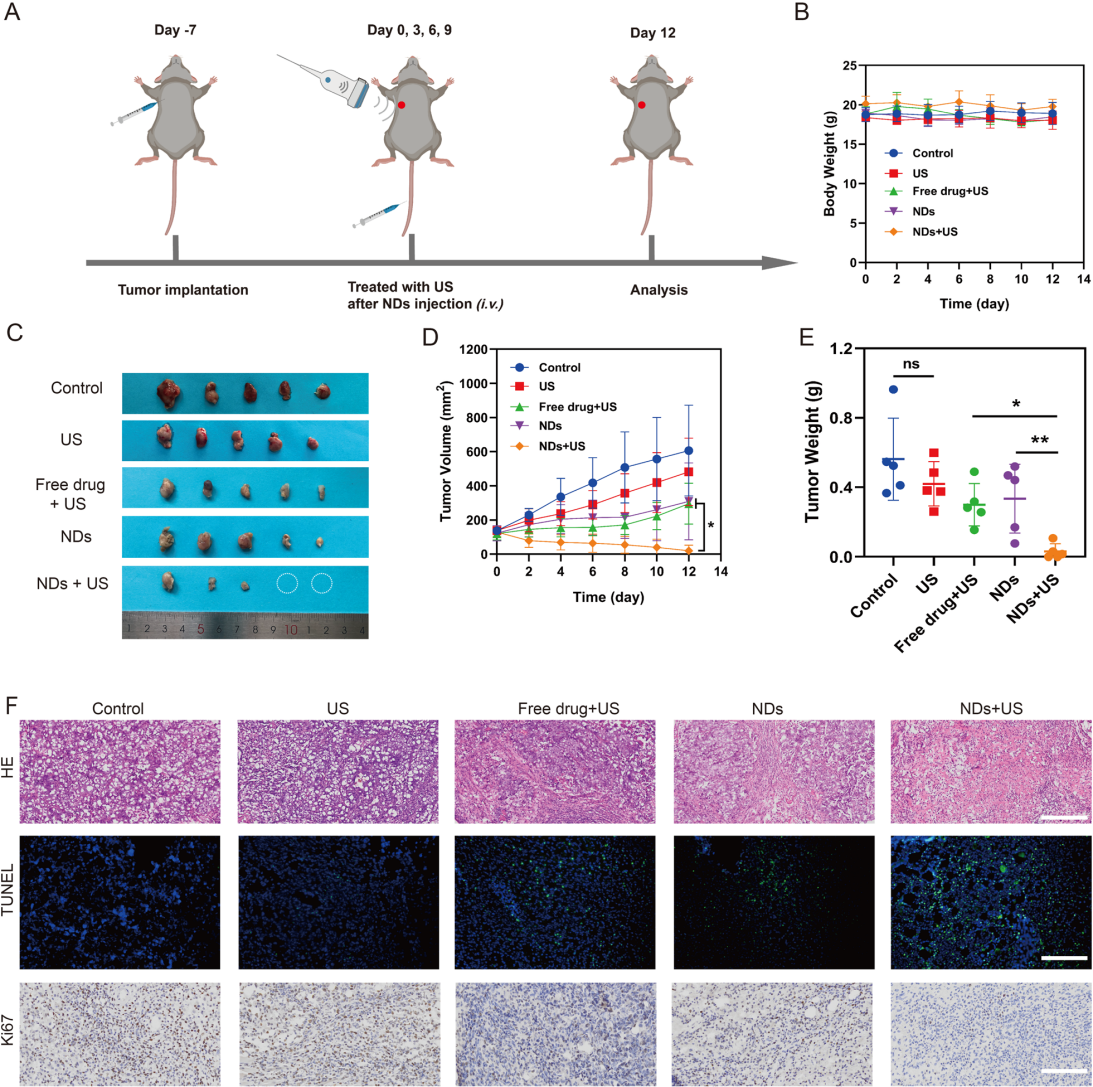
In vivo antitumor effect. **A** Schematic diagram of animal experiment. **B** Body weight of mice at various groups. **C** Photographs of isolated tumors at day 12. The white circle marked that the tumor achieved complete cure during the treatment. **D** Tumor growth in different groups. **E** Tumor weight in each group. **F** HE, TUNEL, and Ki67 staining of tumor sections at the end of experiment. TUNEL positive: green, and cell nuclei stained by DAPI was blue in the fluorescent mode. Scale bar: 200 µm (HE); 100 µm (TUNEL, Ki67). Data are presented as mean ± SD (n = 5, *p < 0.05, **p < 0.01)

To assess the collaborative anti-cancer treatment, mice were randomized into 5 groups. The treatment was performed strictly based on the experimental protocol (Fig. 7A), and tumor volume (Fig. 7D and Additional file 1: S1A) and body weight were recorded every 2 days. Tumors were imaged and weighed on day 12 (Fig. 7C). The tumor volume in ND + US group was remarkably decreased and was found to be the smallest at day 12 (20 ± 29 mm^3^), even two of the five mice achieved complete tumor cure during the treatment period. In comparison, tumor volume was moderately suppressed in the ND group, suggesting that treatment with ND alone was limited in suppressing tumor growth. It could be noticed that inhibitory effect to tumors in US group was not sufficient, demonstrating that ultrasound irradiation alone could not effectively inhibit tumor growth, proving that UTMD played a predominant role in tumor suppression. In addition, tumor volume was smaller in the ND + US group than that in the Free drug + US group (*p < 0.05), indicating that UTMD enhanced the permeability of tumor cell surfaces, promoted the entry of drug into cells and thus evidently improving therapeutic efficacy. Furthermore, in Fig. 7E, compared to the Control and US groups, tumor weights of mice treated with Free drug + US, ND and ND + US for 12 days were remarkably lower, and the smallest tumor weight (0.03 ± 0.04 g) was found in the ND + US group, which was in line with the results of in vitro experiments and strongly proved the effectiveness for tumor therapy.

To further demonstrate antitumor efficacy, histological changes were examined by HE staining. As shown in Fig. 7F, the ND + US group showed obvious nuclear damage and cytoplasmic degradation, suggesting a potent anti-tumor efficacy, as evidenced by the increased positivity (green) of TUNEL assay and down-regulation of Ki67 (brown). These results were consistent with data of tumor volume and tumor weight, further proving a collective anti-tumor effect between drug-loaded NDs-mediated therapy and ultrasound-induced UTMD.

During the study, all mice stayed healthy and had no significant weight loss (Fig. 7B), meaning that there was neither starvation nor disease. And we conducted HE of main organs (heart, liver, spleen, lung and kidneys). No obvious histopathological lesions were observed in main organs (Additional file 1: Figure S1B), confirming that NDs were biocompatible.

### Hypoxia relief and immune response in vivo

**Fig. 8:**
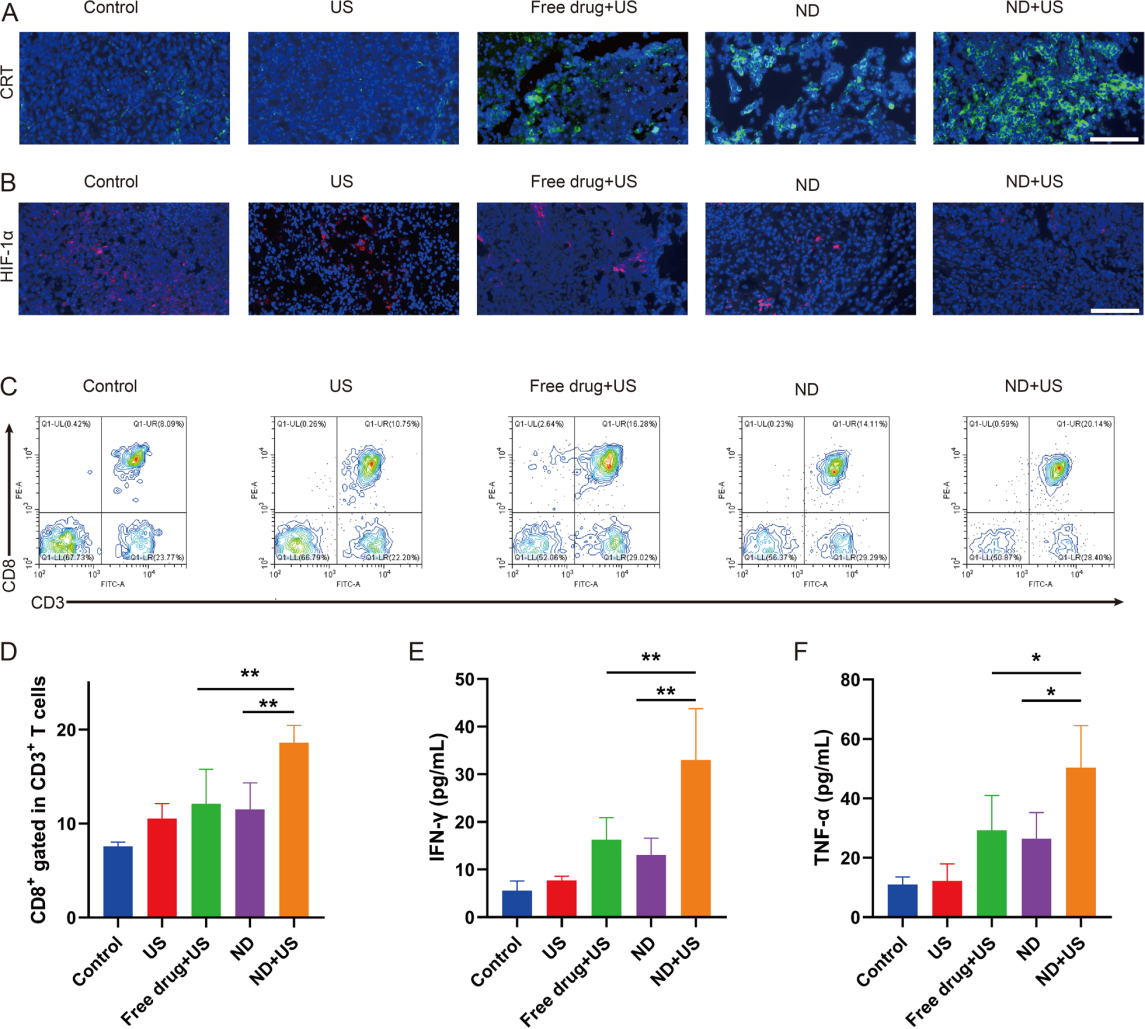
Hypoxia relief and immune response. **A** Immunofluorescence images of CRT of tumor sections in various treatments. CRT marked with DyLight 488-labeled IgG exhibited green fluorescence, and the nuclei labeled with DAPI staining solution exhibited blue fluorescence. Scale bar: 100 μm. **B** HIF-1α expression on tumors of different groups. HIF-1α showed red fluorescence, and the nuclei labeled with DAPI showed blue fluorescence. Scale bar: 100 μm. **C, D** FCM and percentages of CD8 + T cells (gated CD3 +) in tumors. **E** IFN-γ expression level assessed by ELISA. **F** TNF-α expression level detected by ELISA. Data are presented as mean ± SD (n = 3, *p < 0.05, **p < 0.01)

The potent antitumor efficacy in the ND + US group might be ascribed to synergistic effect of chemoimmunotherapy. To investigate antitumor immune response, tumors were collected at the end of treatment and the related immune markers were detected by immunofluorescence staining and FCM assay. CRT was first detected by immunofluorescence. As depicted in Fig. 8A, the least green fluorescence (CRT) was noticed in the Control group. On the contrary, the treatment of NDs combined with US resulted in increased expression of CRT on cell membranes, probably because ROS generated by UTMD combine with PTX released in the NDs could elicit ICD.

Hypoxia in TME leaded it into an immunosuppressed status, which further inhibited T cell infiltration of tumor, decreased the capability of the immune system to treat the tumor and reduced the sensitivity to chemotherapy. NO could downregulate HIF-1α to increase the sensitization to chemotherapy. The HIF-1α in ND + US group was lower compared with the Control, US, Free drug + US and ND groups (Fig. 8B). It was most likely due to the fact that NO generated by NDs through ultrasound promoted the vasodilation and alleviated hypoxia at tumor sites [45]. More importantly, NO could enhance antitumor immunity by increasing the expression of immune factors CD8 + cytotoxic T cells [46]. Figure 8C, D displayed that the US treatment leaded to the mildly infiltration of CD8 + T cells (10.56 ± 1.3%). By contrast, the percentage of CD8 + cytotoxic T lymphocytes (CD3 + CD8 +) in the ND + US group obviously increased to 18.6 ± 1.5%, which was higher than that in the Free drug + US (12.1 ± 2.9%, **p < 0.01) and ND (11.5 ± 2.3%, **p < 0.01) groups. These results proved that the drug-loaded NDs under ultrasound stimulation could enhance T cell infiltration for cancer immunotherapy.

Levels of cytokines, including IFN-γ and TNF-α, were also examined to assess effectiveness of immunotherapy. Similarly, highest secretion of IFN-γ (Fig. 8E) and TNF-α (Fig. 8F) could be measured in the ND + US group compared to several other groups. Apparently, chemoimmunotherapy with NO and PTX provided by NDs under ultrasound irradiation considerably enhanced the anti-tumor immune response.

## Conclusion

In summary, we successfully prepared a novel nanodroplets to co-deliver L-Arg and PTX for enhanced chemoimmunotherapy. Firstly, L-Arg@PTX nanodroplets leaded to significantly alleviated hypoxia and enhanced infiltration of immune cells at the tumor site owing to NO release, which could synergize with chemotherapy induced by PTX and lead to strong antitumor effect, in addition, PTX could induce and amplify ICD, thereby collectively increasing immune response in vitro and in vivo. Unlike other applications of UTMD, it dominated in the release of NO by generating ROS in this study, which was non-invasive and efficient. Moreover, L-Arg@PTX nanodroplets were endowed with endogenous acid and exogenous ultrasound responsive accumulation in tumors, good imaging property and biosafety. Overall, this versatile nanodroplets exhibited their advantages and feasibility in chemoimmunotherapy and provided an exceptional perspective for HCC diagnosis and treatment.

## Supplementary Information

**Additional file 1: Figure S1.** A Tumor volume change per mouse at various treatments. **B **HE staining in main organs after the treatments. Scale bar: 100 µm. n=5. **Figure S2.** NO concentration after being triggered by ultrasound irradiation. n=3. **Figure S3.** **A** Fluorescent images of Hepa1-6 cells treated with DiO-labeled NDs under different pH values at 12, 24 h. NDs labeled with DiO appeared green dots, and cell nuclei counterstained by Hoechst were blue. Scale bar: 50 µm. **B** Quantitative histogram of FCM of Hepa1-6 cells treated with DiO-labeled NDs under different pH values at 12, 24 h. n=3.

## Data Availability

The data of this study is available from the corresponding authors on reasonable request.
