# Supplementary material for: Ultrasound targeted microbubble destruction-triggered nitric oxide release via nanoscale ultrasound contrast agent for sensitizing chemoimmunotherapy
Source: J Nanobiotechnology. 2023 Jan 30;21:35. doi: 10.1186/s12951-023-01776-8 (PMC9885630; doi:10.1186/s12951-023-01776-8)
Supplement: Supplementary file 1 — Additional file 1: Figure S1. A Tumor volume change per mouse at various treatments. B HE staining in main organs after the treatments. Scale bar: 100 µm. n=5. Figure S2. NO concentration after being triggered by ultrasound irradiation. n=3. Figure S3. A Fluorescent images of Hepa1-6 cells treated with DiO-labeled NDs under different pH values at 12, 24 h. NDs labeled with DiO appeared green dots, and cell nuclei counterstained by Hoechst were blue. Scale bar: 50 µm. B Quantitative histogram of FCM of Hepa1-6 cells treated with DiO-labeled NDs under different pH values at 12, 24 h. n=3. [file 12951_2023_1776_MOESM1_ESM.docx]

**Supplementary Materials**

**Additional file 1: Figure S1. (A)** Tumor volume change per mouse at various treatments. **(B)** HE staining in main organs after the treatments. Scale bar: 100um. n=5.


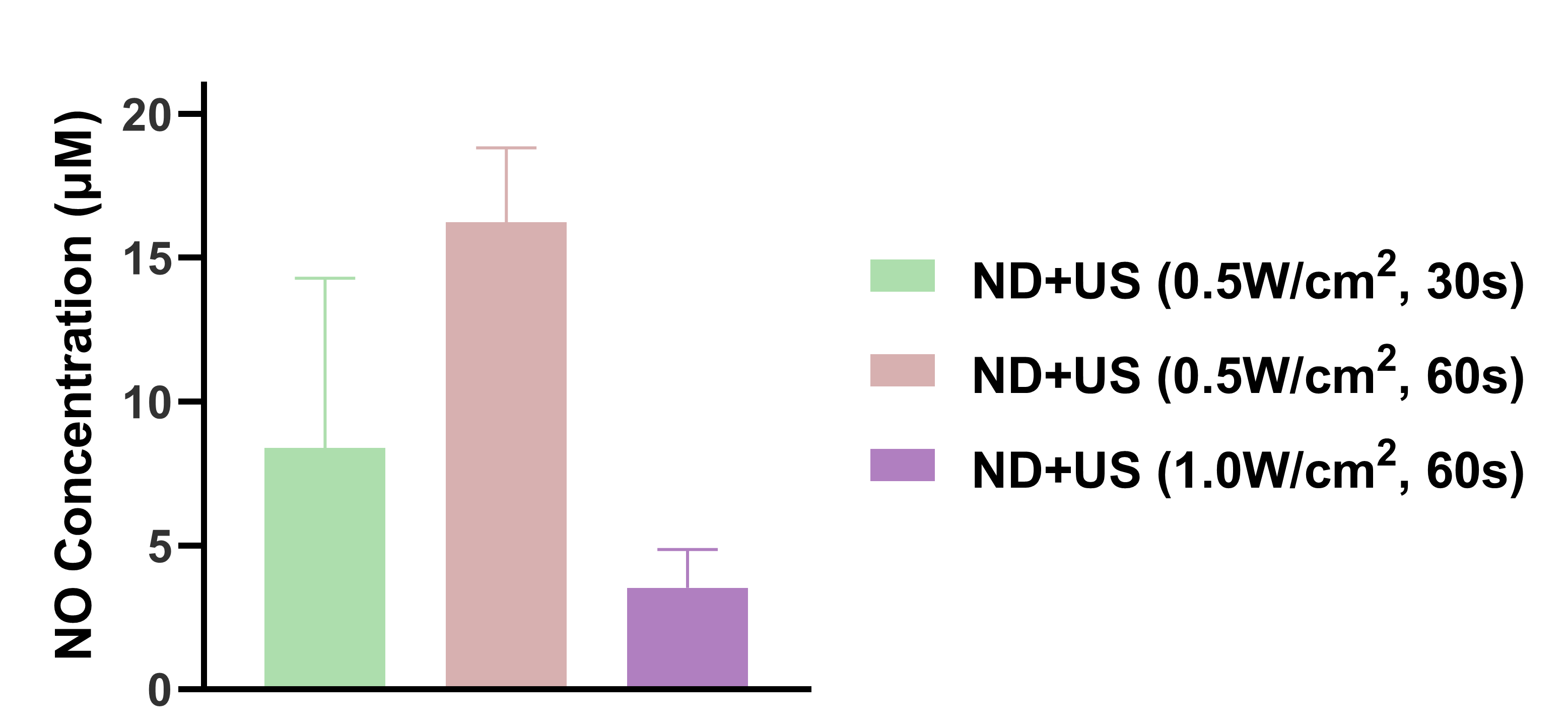


**Figure S2.** NO concentration after being triggered by ultrasound irradiation. n=3.


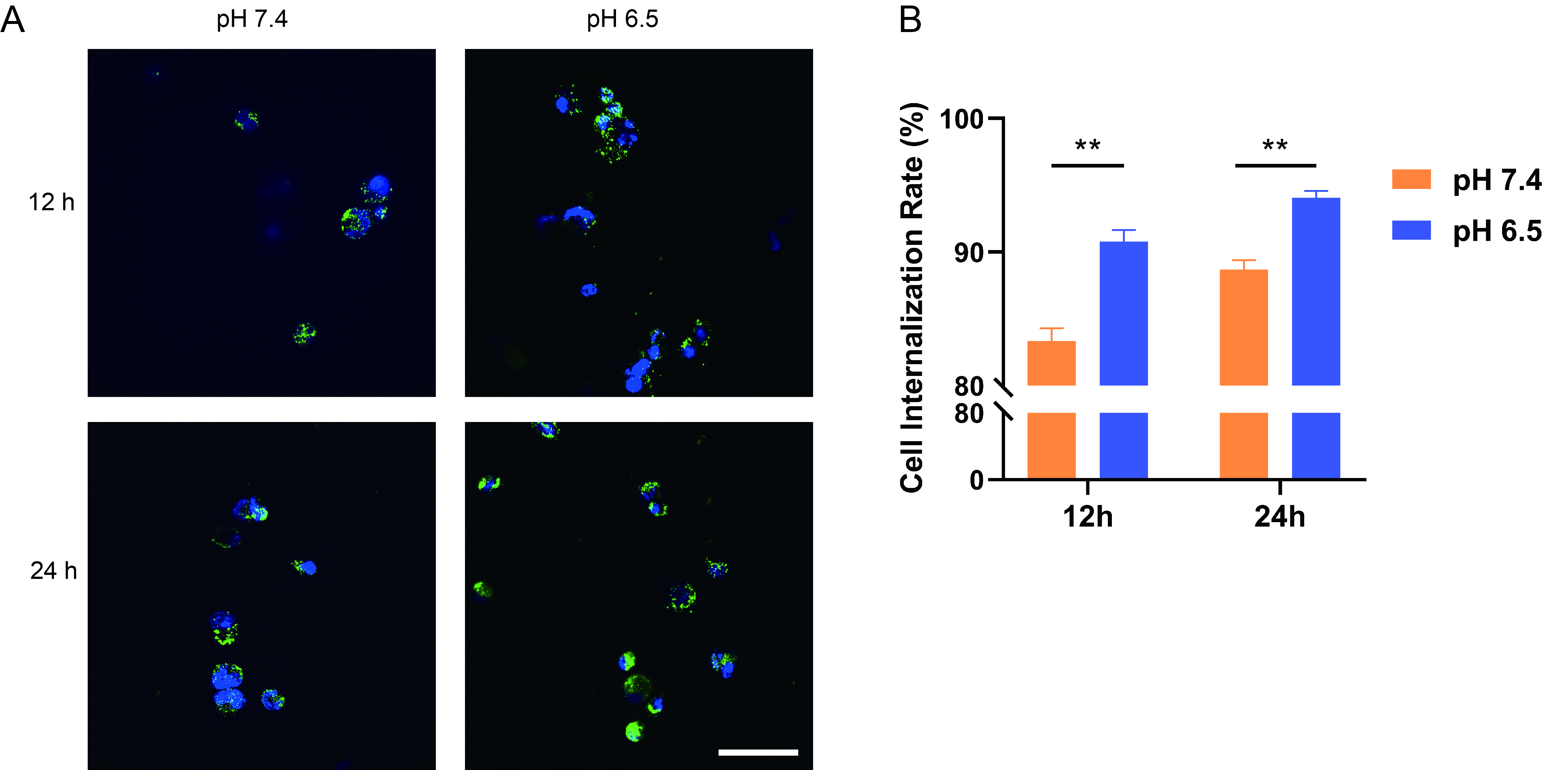


**Figure S3. (A)** Fluorescent images of Hepa1-6 cells treated with DiO-labeled NDs under different pH values at 12, 24 h. NDs labeled with DiO appeared green dots, and cell nuclei counterstained by Hoechst were blue. Scale bar: 50 μm. **(B)** Quantitative histogram of FCM of Hepa1-6 cells treated with DiO-labeled NDs under different pH values at 12, 24 h. n=3.
